# Towards the self and away from the others: evidence for self-prioritization observed in an approach avoidance task

**DOI:** 10.3389/fpsyg.2023.1041157

**Published:** 2023-05-05

**Authors:** Neelabja Roy, Harish Karnick, Ark Verma

**Affiliations:** Department of Cognitive Science, Indian Institute of Technology Kanpur, Kanpur, India

**Keywords:** self-prioritization, approach avoidance, group-prioritization, self-expansion, perceptual matching

## Abstract

Processing advantages arising from self-association have been documented across various stimuli and paradigms. However, the implications of “self-association” for affective and social behavior have been scarcely investigated. The approach-avoidance task (AAT) offers an opportunity to investigate whether the privileged status of the “self” may also translate into differential evaluative attitudes toward the “self” in comparison to “others”. In the current work, we first established shape-label associations using the associative-learning paradigm, and then asked the participants to engage in an approach-avoidance task to test whether attitudinal differences induced on the account of self-association lead to participants having different approach-avoidance tendencies toward the “self-related” stimuli relative to the “other-related” stimuli. We found that our participants responded with faster approach and slower avoidance tendencies for shapes associated with the “self” and slower approach and faster avoidance tendencies for the shapes associated with the “stranger.” These results imply that “self-association” may lead to positive action tendencies toward “self-associated” stimuli, and at the same time lead to neutral or negative attitudes toward stimuli not related to the “self”. Further, as the participants responded to self-associated vs. other-associated stimuli cohorts, these results may also have implications for the modulation of social group-behaviors in favor of those like the self and against those in contrast to the self-group.

## Introduction

Several studies have documented preferential processing for stimuli associated with the “*self*” as opposed to stimuli associated with close-others (friend, mother) or irrelevant others (stranger) and even famous personalities (for a detailed review see Sui and Humphreys, 2015, 2017). Such an advantage for processing self-associated stimuli has been observed for different mental functions such as perception (Sui et al., 2012), attention (Keyes and Brady, 2010; Alexopoulos et al., 2012), memory (Turk et al., 2008; Yin et al., 2019; etc.), decision – making (Polman, 2012), and also, for different kinds of stimuli, for instance, self – names (Moray, 1959; Tacikowski and Nowicka, 2010), self – faces (Brédart et al., 2006; Keyes and Brady, 2010; Woźniak and Hohwy, 2020), self-owned objects (Cunningham et al., 2008; Constable et al., 2019).

More recently, Sui et al. (2012) have demonstrated that processing advantages can also be obtained for arbitrary geometrical shapes (e.g., square, circle, triangle.) once they have been associated with socially salient labels such as “self”/ “you” in comparison with other labels such as “friend,” “stranger” or even “mother” (Sui and Humphreys, 2017). The paradigm used by Sui et al. (2012) has been referred to as the associative – learning paradigm and has been utilized to demonstrate the advantages for self-association, across a range of tasks, and as distinguishable from the effects of semantic elaboration (Sui and Humphreys, 2013), effects of prior familiarity (Sui et al., 2014) and even reward allocation (e.g., Schäfer et al., 2016; Stolte et al., 2017).

Given the findings, that the advantages of self-association can be obtained by momentarily associating arbitrary geometric shapes with socially salient labels such as “self” or ‘you” (Sui et al., 2012), irrespective of the stimuli’s prior familiarity with the participants, researchers have proposed that merely encoding a particular stimulus in relation to one’s own self may be sufficient to give rise to benefits in subsequent processing (Kim et al., 2018; Macrae et al., 2018). Indeed, Sui and Humphreys (2015) have argued for a special role of self–association in the processing of perceptual information and suggested that it may function as an integrating influence for the different stages of information processing across perception, memory, and decision - making.

Previous research has also established that the relative superiority for stimuli associated to the self can be extended to group-memberships as well. For instance, Moradi et al. (2015) demonstrated preferences toward participants’ favorite football team, whereas Enock et al. (2017) demonstrated preferences for members of own team versus those of a rival or a neutral team. These results put together can be taken to imply that the emergence of the advantage associated with “self-referential” processing may in part arise due to the definition of the self, relative to others, as is clear from the first body of studies described above.

Such a proposal is in line with suggestions such as Knight (2015) wherein he has argued that one’s definition for the “self” could be emergent from the recognition of contrasts and difference with others. Knight (2015) further adds that such a process of self-definition in the context of the “other” could also render negative consequences such as over identification with the in-group, while facilitating negative behavioral outcomes for those not belonging to the in-group (Knight, 2015).

One of the most important aspects of social behavior is the formation of attitudes and their consequent manifestation in the form of action tendencies toward “attitude objects.” Indeed, several researchers have expressed the definition of “attitudes” as predispositions of behavior toward objects, events, or people in their external environment. For instance Bogardus (1931) defines, attitudes as “a tendency to act toward or against something in the environment which acquires thereby a positive or a negative value (p.62).” (as quoted in Chen and Bargh, 1999). Similarly, Allport (1935) defines attitudes as a “mental state of readiness…exerting a directive or dynamic influence upon the individual’s response to all objects and situations with which it is related.” (p. 80) [as quoted in Chen and Bargh (1999)]. Also, attitudes have been deemed to play an important role in an individual’s interaction within a social situation, as Triandis (1989) aptly defines attitudes as “an idea charged with emotion which predisposes a class of actions to a particular class of social situations” (p.2) [as quoted in Chen and Bargh (1999)].

Further, Chen and Bargh (1999) propose that individuals are constantly involved in subconsciously/automatically evaluating objects or situations that they encounter and that this automatic evaluation manifests in preparing appropriate responses (positive/negative or approach/avoid) toward stimuli, in congruence with the results of the evaluation.

Such a proposal from the authors is in line with previous claims, such as Lewin (1935) assertion that objects and events from the external environment automatically acquire valences that determine behaviors toward them. It seems that the process of evaluation is intrinsic to individuals’ interaction with the environmental objects or events. For instance, Osgood et al. (1957) claimed that such an evaluation was a major component of the semantic meaning of an object and served as a guide for behavior toward a given object, and Lang et al. (1990) contended that the mere presence of an object automatically results in the activation of either a positive approach or a negative avoidance tendency depending upon stimulus valence.

Indeed, Chen and Bargh (1999) have demonstrated that their participants were faster to respond to negatively valenced stimuli with an avoidance response (pushing the lever away) and to positively valenced stimuli with an approach response (pulling the lever toward themselves).

Recently, Lavender and Hommel (2007) deriving from the theory of event coding (TEC: Hommel et al., 2001) have contested the proposal and opined that approach-avoidance responses may simply follow from more general mechanisms of action control, not specific to valence processing. More specifically, they proposed that approach-avoidance responses result from an overlap between the valence of the stimulus and the valence of the response.

While a more detailed discussion of the varying accounts of the approach-avoidance behavior may be beyond the scope of the current work, it must be noted that the elicitation of the approach-avoidance response has been used as an indication of the participants’ evaluation of the different “attitude objects” across various versions of the so-called Approach-Avoidance Task (AAT).

To describe the Approach Avoidance Task in more detail, it typically involves participants’ evaluating a range of affective stimuli and making approach-avoidance responses toward them (for a detailed discussion of various types of tasks see Phaf et al., 2014). A common finding is that the participants are faster at making approach responses and slower at making avoidance responses toward positively evaluated stimuli, while at the same time they are found to make faster avoidance responses and slower approach responses toward negatively evaluated stimuli (Phaf et al., 2014). These findings have been consistently reported in different versions of the approach avoidance task, for instance, the original lever task (Chen and Bargh, 1999), the manikin task (De Houwer et al., 2001) or the feedback-joystick task (Rinck and Becker, 2007). Although, a closer look may suggest that the different versions of the approach-avoidance task may differ in their sensitivity toward tapping the effects of valence on the approach-avoidance tendencies of individuals (Krieglmeyer and Deutsch, 2010; Phaf et al., 2014).

Several studies have demonstrated that positively evaluated stimuli spontaneously facilitate positive approach actions while negative stimuli trigger aversive or avoidance tendencies, in the different versions of the AAT task (Chen and Bargh, 1999; Wentura et al., 2000; Markman and Brendl, 2005; Rinck and Becker, 2007; Seibt et al., 2008). For instance, Klein et al. (2011), used the AAT to evaluate automatic behavioral tendencies in children, in response to spider pictures as opposed to pictures of butterflies or neutral pictures, and found that the pictures of the spiders elicited avoidance responses, whereas those of the butterflies elicited approach responses, in comparison to neutral pictures. Similarly, Galler et al. (2022) demonstrated that children’s approach biases as tested in their AAT task were closely related to their likeness ratings for sweet and high calorie snacks, and in general toward food pictures in comparison to non-food pictures. Wittekind et al. (2021) went a step further and compared the effectiveness of different response devices for implementing the AAT to assess the behavioral tendencies toward chocolate and found their participants to be having a strong approach bias toward chocolate regardless of the response device (i.e., joystick, computer mouse and a touchscreen).

Various versions of the AAT task have also been employed to study individuals’ response tendencies toward different social situations and group dynamics as well. For example, Paladino and Castelli (2008), reported that people were more efficient in approaching the photos of members of their ingroup and avoiding the outgroup (based only on the color of the adhesives). Similarly, using the Visual Approach/Avoidance by the Self Task (VAAST), Rougier et al. (2020) have demonstrated that despite the across-participant variabilities in intergroup attitudes, both non-dominant as well as dominant groups exhibited a significant ingroup bias.

Besides the implementation of the AAT paradigm in lab-based studies, several researchers have used web-based/online, mobile based and even virtual – reality based AAT paradigms. For instance, Weil et al. (2017) reported that an online intervention with the approach-avoidance task was instrumental in their participants’ reduction of OCD symptoms. In the same vein, Macy et al. (2015) employed a web-based approach – avoidance intervention and demonstrated that the implicit attitudes of smokers could be modified based on their exposure to information modulating their attitudes about smoking.

Based on the above findings, it can be safely assumed that the Approach – Avoidance Task (AAT) may be useful to investigate the evaluation of individual’s attitudinal responses toward “self-related” stimuli in comparison to the “other-related stimuli.” Interestingly, a recent study by Barton et al. (2021) also sought to explore individual’s approach – avoidance tendencies using button-press approach – avoidance responses, in response to self-owned vs. experimenter owned objects. The authors observed a significant self-bias in the initiation time for responses toward the self-owned mug and also for overall movement duration, however, the effects got attenuated when the participants were required to respond in an orthogonal task. The authors concluded that ownership status judgments and affective evaluation may involve different mechanisms.

## Experiment design

The experiment consisted of 5 stages, each followed by the other in a sequential manner. The first stage, i.e., the *familiarization stage-I* familiarized the participants to the idea of the manikin and help them get familiar with keypresses to move the manikin upwards and downwards. In the second stage, i.e., *familiarization stage – II*, the participants were familiarized with the concept of approach and avoid as in the AAT paradigm, by approaching and avoiding using a manikin in response to text instructions. In the third stage, i.e., *association – I*, participants were instructed to associate the colored shape combinations with social labels, “you” and “stranger.” The labels “self” and “stranger” were associated with a small group (3 exemplars) of stimuli that were a combination of features, e.g., “self” could be associated with “a cyan triangle” and typically they would be presented with three unique exemplars of the kind of shape (i.e., cyan triangle) and would be required to make approach – avoidance responses toward 10 new randomly chosen instances of the same shape-color combinations. In the fourth stage, i.e., *association – II*, the established associations were tested through a shape-label matching task (described in detail below) to ascertain that the associations between the shapes and labels were indeed formed. The fifth stage, i.e., *the experimental stage* was where the participants approached or avoided colored-shape combination using the movements of the manikin).

The experiment employed a within-subjects factorial design. First we carried out a 2 × 2 × 2 repeated measures ANOVA with Stimulus Category having two levels (Self and Stranger), Action Type having two levels (Approach and Avoid) and Manikin Position having two levels (Bottom and Top). Next we carried out a 2 × 2 repeated measures ANOVA, where 2 factors with 2 levels were manipulated, i.e., Stimulus Category having two levels (Self and Stranger) and Action Type having two levels (Approach and Avoid), as within subjects variables. Hence for analyses, we used a 2-way within subjects repeated measures ANOVA to look into the main effects of Stimulus Category, Action Type and their interaction, i.e., Stimulus Category × Action Type. *Post-hoc* comparisons were also conducted to look into individual variable effects, like the differential effects of Stimulus Category (Self vs. Stranger) and Action Type (Approach vs. Avoid).

We purposely kept only two socially salient labels in contrast because (a) the two labels, i.e., “self” and “stranger” were most distinct from each other in terms of the difference in perceptual performance induced in terms of social saliency (Sui et al., 2012; Verma et al., 2021). Here, we expected to see differences in approach vs. avoidance tendencies in the “self” vs. “stranger” labels, as in previous work it has been demonstrated that in the case where participants were asked to make match/unmatch responses to a bunch of exemplars associated with the socially salient labels, the responses were most clearly distinct for “self” in comparison to ‘stranger” associated stimuli (see also Stein et al., 2016; Vicovaro et al., 2022).

## Materials and methods

### Participants

Based on the number of participants used in previous studies employing the manikin based approach avoidance task (e.g., Saraiva et al., 2013) and shape – label association task (Sui et al., 2012), we decided to keep the sample size at 36 participants. For the sample size of 36 participants, a power analysis according to G*power (Faul et al., 2009), showed that a modest effect size of 0.2, could be detected with power (1 – ß) = 0.8 and alpha = 0.05. While 36 participants took part in the experiment, the data from only 34 right-handed participants (range 18−26 years, mean age = 22.8 years, SD = 1.62 years; 31 M, 4 F) with normal or corrected-to-normal vision, were analyzed. One datapoint was lost owing to a technical glitch, and one participants’ data had to be removed as the average response times for this participant were less than 100 ms, indicating erroneous responding patterns. Informed consent was obtained from all the participants and they were duly compensated for their efforts. Prior approvals were obtained duly from the Institute Human Ethics Committee at Indian Institute of Technology, Kanpur.

### Procedure

**FIGURE 1:**
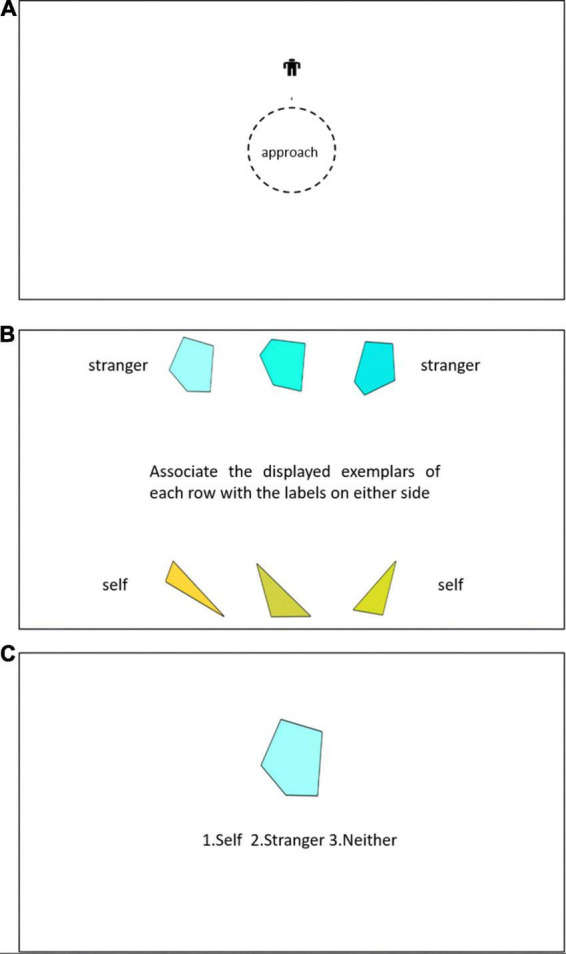
The flow of the **(A)** Stage-II, **(B)** Stage-III, and **(C)** Stage-IV of the experiment.

The experiment was conducted in 5 phases. In the first phase i.e., the *familiarisation phase -I*, to accustom the participants to the manikin and its movement, participants were required to move a manikin i.e., representing as a schematic image of a human figure up and down an empty screen by pressing keys “Y” and “B,” respectively, for a short span. Following previous Manikin-based studies, participants were encouraged to imagine that the manikin was an on-screen representation of themselves (Saraiva et al., 2013) and thus imagine moving along with the manikin. The manikin was first presented for 2 s after which the participants could practice the movement of the manikin by pressing the B and Y keys for about 1−2 min. In the second phase i.e., *familiarization phase – II*, participants were introduced to an instance of the approach-avoidance task, for 12 trials. Here, participants responded to text-instructions of “approach” or “avoid.” In essence, after a fixation cross had been presented for a variable duration of 500−1,000 ms, a manikin was then presented above or below the text prompt for 500 ms with equal probability and it stayed on while the participants had to move the manikin closer or farther from the text by three consecutive presses of the required key (either “Y” or “B”) in the required direction as indicated by the text instruction (see Figure 1A). This paradigm of approach and avoidance was the same as in the main phase (phase V) of the experiment as described next, except that a text instruction appeared and stayed on the screen till response completion, instead of the colored shapes. In the third phase, i.e., *the association phase - I* (see Figure 1B), the participants were shown a screen where sets of 3 colored shape exemplar stimuli was presented in two rows along with their associated labels, i.e., triangles – you, pentagons – stranger (counterbalanced across participants). They were instructed to associate and encode the colored-shapes, drawn from the two exemplar categories and pair them with either of the social labels “Self” or “Stranger.” The instructions remained on the screen till the participants pressed a spacebar and moved on to the next screen. Next, in the fourth phase, i.e., *association phase - II*, to ensure that participants remembered the associations, an association-matching task was conducted. In this task, participants were first presented with a blank screen for 1 s, followed by a fixation cross (black on white background) for 500−1,000 ms. After the fixation cross, participants were presented with a colour shape combination on top of the fixation cross and a label “you” or “stranger” below the fixation cross. The stimuli stayed on the screen until the participants responded by pressing either of 1, 2, or 3 number keys that indicated that the presented color – shape combination matched with either 1: self, 2: stranger, or 3: neither (see Figure 1C). There were 18 trials for each participant in the association task, and accuracy was used as criteria for whether the participant could move to the next stage. The overall accuracy for participants was around 91% (*M* = 91.26, SD = 8.4).

**FIGURE 2:**
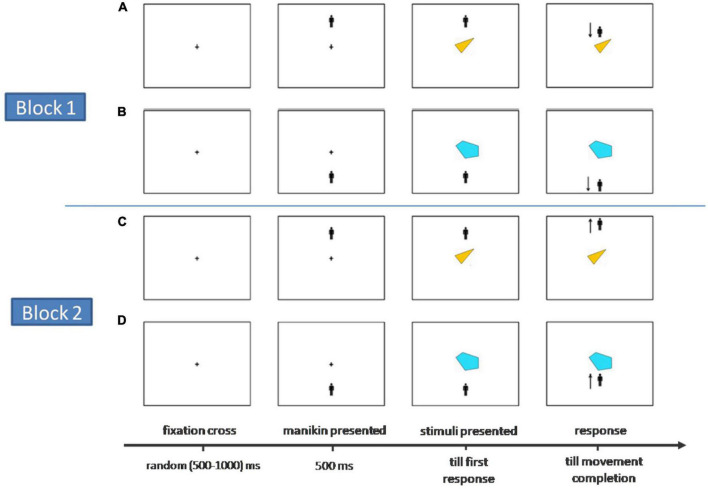
The flow of the approach-avoidance movements with the manikin in Stage-V of the experiment. Block 1: Self-matched exemplars approached, and Stranger avoided. Block 2: The reverse condition. **(A)** Trials where participants approach colour-shape stimuli of Self with manikin at top. **(B)** The Stranger-associated stimuli where participants avoid it with manikin presented below the exemplar in the lower half. **(C)** Participants required to avoid Self-associated colored-shapes. **(D)** Participants approach Stranger-associated.

In the fifth and final phase, i.e., the Manikin – Based Approach Avoidance task, participants were instructed, as in Rougier et al. (2020), to use only the index finger of their dominant hand for the purpose of keypress (“Y” or “B”) to make the required approach and avoidance movements in the task. All trials were supposed to start when participants would bring their finger to press the “H” key which lies in between the “Y” and ‘B” keys in the standard QWERTY keyboard. This was hoped to ensure that the participants brought their finger back to a neutral point after executing a response keypress so that they can respond to the next keypress without any positional inertia of their fingers. The trial started after the keypress of H, with a fixation cross (0.5 × 0.5 cm) appearing at the center of the screen for a randomly chosen interval between 500 to 1,000 ms, which was followed by the manikin (1.25 × 1 cm) appearing either above or below the fixation cross (6 cm away) with equal probability for 500 ms, following that, the participants were presented with a randomly chosen colored shape stimulus (4.5 × 4.5 cm) at the center of the screen. Now, they had to either move the manikin closer (approach) to the target stimulus or move away (avoid) depending on their perceptual categorization of the given stimulus (say, approach if self-exemplars and avoid if stranger, or opposite). Participants were encouraged to try to move as fast as possible and the RTs were recorded as the duration between the onset of the stimulus to the first correct keypress toward the desired direction as per the required condition. The manikin could appear in either the lower or upper half of the screen and the participants had to respond by keypresses (“Y” or “B”) to move the manikin up or down to approach or avoid. The visual stimuli persisted till the movement ended with three consecutive keypresses in the correct direction. The experiment comprised two blocks, in one block participants had to avoid the shapes which have been associated with “Self” and approach those associated with ‘Stranger” while in the next block they had to do the reverse. The order of the presentation of the stimuli was randomized and the order of the blocks was counterbalanced across participants (see Figure 2 for the graphic depiction of the flow).

The main test block started after completion of the practice trials, which lasted for 12 trials in each block, where a very faint destination marker aided the participants in indicating where exactly to reach while approaching or avoiding. No such markers were available during the testing trials. The conditions thus were Self-approach, Self -avoid, Stranger-approach, Stranger-avoid. There were 120 trials in all in the testing block for each participant with 30 trials for each condition and the two blocks, Self-Approach with Stranger-Avoid and Stranger-approach with Self-Avoid of 60 trials each, separated by a sufficient time of break. The number of trials used is in line with studies using AAT tasks (Chen and Bargh, 1999; Krieglmeyer et al., 2010).

### Stimuli and medium

**FIGURE 3:**
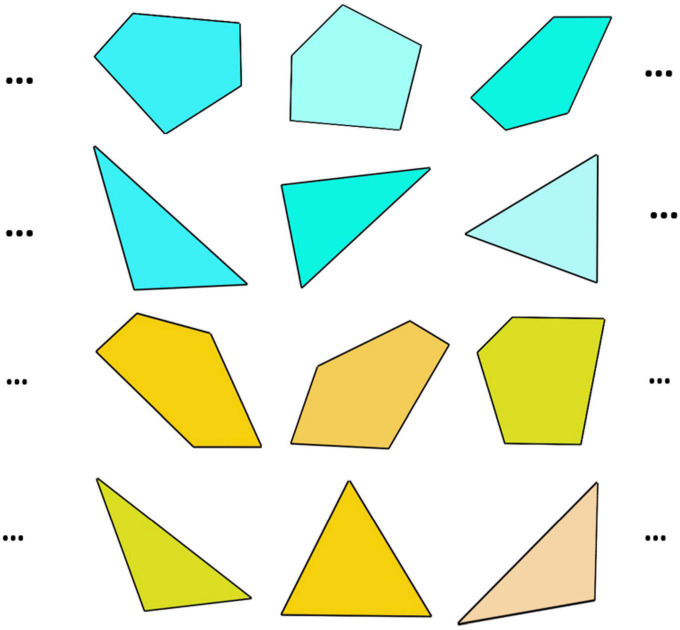
Families of colored-shape exemplars of triangles and pentagons of colors cyan and yellow a limited subset of which has been familiarized with associations while the AAT task is then performed on a much larger collection of exemplars, even novel ones, sharing the same two defining features of an exemplar category.

The experiment was coded using Psychopy and Python 3.7, and was conducted online over the internet using Pavlovia. The target stimuli were a combination of colors and shapes, wherein the different shades colors were chosen from two categories, i.e., cyan and yellow and the shapes were from different variations of triangles (number of vertices = 3) and pentagons (number of vertices = 5) generated using MATLAB. Groups of 10 such exemplars randomly drawn from each of the color-shape combination categories ((10 such cyan triangles and 10 yellow pentagons) or (10 yellow triangles and 10 cyan pentagons)) were utilized for the experiment, counterbalanced across participants. We used a combination of color and shape as stimuli, following from a previous study, where we were able to demonstrate that participants were capable of handling a more complex characterization of the arbitrary geometrical shape” as used in Sui et al. (2012). A sample of the possible colour-shape combination of stimuli is shown in Figure 3. The colour – shape combinations and their associations to the labels of “Self” or “Stranger” were selected randomly across the participants. The participants undertook the experiments on their laptops or workstations placed on a stable surface like a table in an isolated, silent and dark room. The execution of the task by the participant was constantly monitored through the “Share Screen” feature with due permission over video conference platforms like Zoom/Skype to ensure that the communicated instructions are being received and correctly followed.

## Results

The analyzes were conducted using JASP, a free open-source software (JASP Team, 2023). For the analyses, we used the response times of all the first correct keypresses that initiated the movement of the manikin towards either of the correct directions by the participants. We have used the average RT of the first correct keypresses, as a dependent variable (De Houwer et al., 2001) to measure response differences across participants. Also, to avoid momentary inattention and anticipatory responses affecting our results, we eliminated responses that were faster or slower than two standard deviations from the mean RTs, for each participant in each condition.

Before the overall analyses, we wanted to look into whether the initial starting position of the manikin, i.e., whether it started on the top or below the center of the screen had any impact on participant’s responses. For this purpose, we carried out a 2 × 2 × 2 within- subjects repeated measures ANOVA with Stimulus Category (Self, Stranger) × Movement Type (Approach, Avoid) × Position of Manikin (Top, Bottom) as the three within-subjects factors. Here we did not find a significant main effect of Stimulus Category, *F*(1,33) = 0.094, *p* = 0.761, $\eta_{p}^{2}$ = 0.003. However, we found a significant main effect of Movement type (Approach vs. Avoid), *F*(1,33) = 6.654, *p* = 0.015 (<0.05), and the main effect of Manikin Position (Above, Below), *F*(1,33) = 9.770, *p* = 0.004 (<0.05), $\eta_{p}^{2}$ = 0.228.

Amongst the interaction effects, only the interaction between Category × Movement, *F*(1,33) = 14.338, *p* < 0.001, $\eta_{p}^{2}$ = 0.303 was found to be significant, while the interaction between Category × Manikin Position, *F*(1,33) = 1.338, *p* > 0.05 ( = 0.256), $\eta_{p}^{2}$ = 0.039; Movement Type × Manikin Position, *F*(1,33) = 0.448, *p* > 0.05 ( = 0.508), $\eta_{p}^{2}$ = 0.013 and the interaction between Category × Movement Type × Manikin Position was not found to be significant *F*(1,33) = 0.765, *p* > 0.05 ( = 0.338), $\eta_{p}^{2}$ = 0.023.

Since, the interaction of Manikin Position (Above vs. Below) with the other two variables, i.e., Target Category and Movement type was not found significant, we concluded that the initial position of the manikin was not a moderating factor for our overall results. All subsequent analysis use collapsed RTs across the two manikin positions (above vs. below) for each target category × movement type.

**FIGURE 4:**
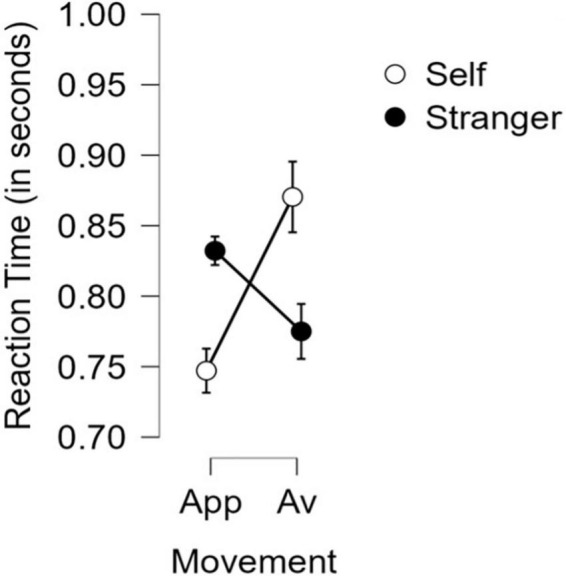
Approach (App) and Avoidance (Av) measures for the group of Self and Stranger associated exemplars along with their Standard Error bars.

We found that the participants were faster for all approach responses as compared to the avoidance ones, *t* = −2.579, p_*holm*_ = 0.015, Cohen’s *d* = −0.115. Through more detailed *post-hoc* comparisons with the Category (Self, Stranger) × Movement Type (Approach, Avoid) interaction, we found that the participants were much faster to approach the ‘self” than to avoid it, *t* = −4.557, p_*holm*_ < 0.001, Cohen’s *d* = −0.506; and they were also much faster to approach the “self” than to approach the “stranger,” *t* = −2.915, p_*holm*_ = 0.020, Cohen’s *d* = −0.349. On the other hand, even though they were not significantly slower to approach the stranger than to avoid it, *t* = 2.114, p_*holm*_ = 0.118, Cohen’s *d* = 0.235, they were indeed significantly much slower to avoid the self than to avoid the stranger, *t* = 3.452, p_*holm*_ = 0.009, Cohen’s *d* = 0.392. The mean RT data for the experiment is in Table 1. For a graphic representation see Figure 4, for response times aggregated across manikin positions.

**TABLE 1:** Mean and SD of reaction times for approach and avoidance.

Action	Position	RT for self (in milliseconds)		RT for stranger (in milliseconds)	
		**M**	**SD**	**M**	**SD**
Approach	Above	786	251	844	216
	Below	708	202	821	246
Avoid	Above	885	294	793	235
	Below	856	288	757	199

M, mean; SD, standard deviation; RT, reaction time.

Overall, we observed that across the trials and participants, the self-related exemplars have been approached with significantly faster RTs and avoided with significantly slower RTs in comparison to stranger related exemplars. Also, self - related exemplars were approached significantly faster than they were avoided.

Moving further, to better understand the explicit approach-avoidance tendencies for the two groups of stimuli related to the self or the stranger, we followed the method proposed by Degner et al. (2016) and others, and calculated the AAT score through the difference between the mean latencies of avoidance and approach reaction times (thus AAT Score = Avoidance RT - Approach RT) separately for each of “self - and “stranger – related” stimuli” as AAT scores for every condition. Please note, when the AAT scores are higher (and positive, meaning more time to avoid and lesser time to approach), they indicate a relative prevalence of approach tendencies as compared to avoidance bias for that particular stimuli or class of stimuli. The observed scores (in seconds) and the corresponding SD values are computed with scores from all approach and avoidance actions (irrespective of the position) and presented in Table 2.

**TABLE 2:** AAT scores for self and stranger categories.

AAT category	AAT score	
	**M**	**SD**
Self	0.118	0.169
Stranger	−0.057	0.109

M, mean; SD, standard deviation.

**FIGURE 5:**
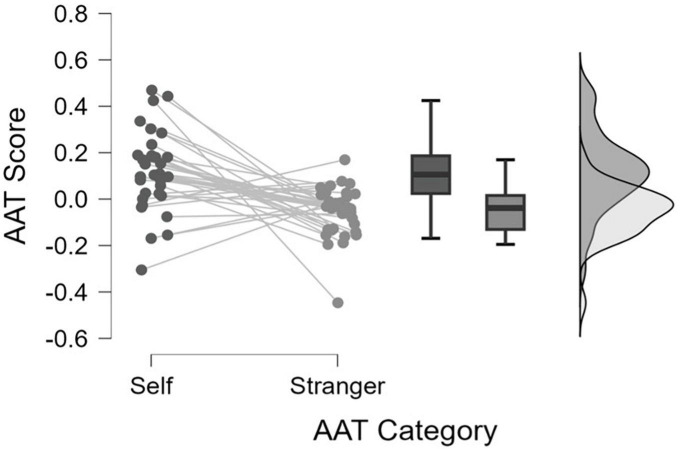
Raincloud plot depicting the differences in AAT Scores across Self-Stranger categories, the Mean, Median, Confidence Intervals and the probability distributions.

Evidently, the AAT score for self-related stimuli was significantly more positive than that for stranger-related stimuli, t(34) = 4.143, *p* < 0.001, Cohen’s d = 1.232 (see also Figure 5), indicating significantly higher approach tendencies for “self-related” stimuli as compared to the stranger-related stimuli.

Moreover, we found that the AAT score for the self-related stimuli was significantly greater than 0 and thus significantly positive, t(34) = 4.083, *p* < 0.001, Cohen’s d = 0.700, whereas the AAT scores for stranger – related stimuli were AAT scores were significantly lower than 0, and thus significantly negative t(33) = −3.059, *p* = 0.002, *d* = −0.522. Overall, once again we observe a significant approach bias toward the self – related stimuli and definite avoidance bias for the stranger – related stimuli.

## General discussion

In the current study, we sought to investigate whether the privileged processing of stimuli associated with the “self” may translate into differential evaluative attitudes toward self-associated stimuli in contrast to stimuli associated with an irrelevant “stranger.”

To achieve the same, we first asked our participants to associate the labels, “self” and “stranger” with three exemplars that were colour-shape combinations (e.g., yellow triangles or cyan pentagons) and remember the same (in phases 3 and 4). Then, we asked our participants to engage in a manikin-based approach avoidance task (De Houwer et al., 2001) (in the fifth phase), wherein they were asked to press specified keys to move the manikin toward (approach) or avoiding exemplars from sets of colored shapes containing even new (previously unseen) exemplars i.e., colored shapes similar to the exemplars they had associated with the “self” or the “stranger” labels. The participants were instructed to imagine that the manikin represented themselves, and that they were moving along with the movements of the manikin.

Previous work has shown that participants are able to generalize the self/stranger-associations to stimuli from a small set of stimuli that they initially form the associations with to a larger cohort of new (previously unseen) stimuli.

Given these findings, we expected that our participants will be able to generalize their response tendencies for the “self” and “stranger – related” stimuli in the (3rd and 4th phases of the study) to the cohort of stimuli resembling the self- and the stranger-related stimuli, during the approach – avoidance task that they participated in the 5th phase of the experiment.

Indeed, as expected our participants demonstrated significantly faster approach movements toward the cohort of “self-related” stimuli as compared to the set of “stranger – related” stimuli, and significantly faster avoidance movements toward the cohort of “stranger – related” stimuli as compared to the cohort of stimuli related to the “self.” Further analyses in terms of the AAT scores, also revealed existence of a clear approach – bias toward the cohort of self-related stimuli and a significant avoidance – bias toward the stranger – related stimuli.

Our results are in line with recent work by Barton et al. (2021), who have reported a significant self-bias in the favor of self-owned objects (i.e., mugs in their experiment) as opposed to objects owned by the experimenter, and similar studies where the approach – avoidance task has been used for self-owned vs. other owned objects (Constable et al., 2011, 2014).

Given, that the Approach Avoidance Task (AAT) taps into the attitudinal evaluation for the stimuli at hand, it would make sense for the participants to show exaggerated approach tendencies and slower avoidance responses for stimuli rated more positively and relatively faster avoidance tendencies and slower approach tendencies for those rated as less positive (e.g., Chen and Bargh, 1999; Krieglmeyer et al., 2010).

Previous research suggests that participants are inclined to treat information referring to themselves as positive. For instance, in a couple of studies (Rogers et al., 1977; Kuiper and Rogers, 1979) participants rated the personality attributes ascribed to the self as more positive and even recalled them better. Also, Greenwald (1980) presents a detailed account of pervasive positivity bias when it concerns the self and self-relevant information. These cognitive biases, according to Greenwald (1980) permeate different aspects of our evaluations of the world, organization of information in memory, in the analyses of cause and effect and so on. Barton et al. (2021) attribute this self-positivity bias as described by Greenwald as one of the factors responsible for speeded responses for self-relevant information in contrast to other types of information. More recently, researchers (Constable et al., 2011, 2014 and Barton et al., 2021) have also speculated that ownership or in other words identification with items as belonging to the self or the other (e.g., a stranger) may itself be an instantiation of positive or negative evaluation for items. More specifically, it has been proposed that items owned by the self might be processed as more positive as compared to items owned by someone else (Barton et al., 2021). It has been suggested that the positive evaluation of self-related items is distinct from a financial or monetary value, and that they are intrinsically rewarding (Sui and Humphreys, 2015). Other studies have also demonstrated the fact that self-association selectively enhances the processing of positive information. For instance, Durbin et al. (2017) demonstrated that self-referential processing enhances source memory for positive words, as opposed to neutral or negative words. Similarly, Watson et al. (2007) showed that their participants responded faster to positive self-referential words as compared to negative non-self-referential words.

Given that information associated with the self is positively evaluated and that the approach – avoidance task (AAT) taps into the affective evaluation of stimuli and the consequent response tendencies associated with the same, it is not surprising that our participants show a significant approach bias toward stimuli related to the self as compared to stimuli related to the stranger (e.g., Chen and Bargh, 1999; Koch et al., 2008; Krieglmeyer et al., 2010).

However, there are a few caveats to our results that must be discussed to understand the results more clearly. For instance, previous research suggests that the approach – avoidance actions during the AAT tasks are also modified by the context in which they occur (Eder and Rothermund, 2008; Eder and Klauer, 2009), wherein it is understood that the behavior could be modulated by the demands from the environment as to whether it requires the individuals (say in form of the manikin) to move away or toward the attitude object like self-related stimuli (i.e., self – reference) or to move the attitude object toward or away from us (i.e., object-reference). Researchers have discussed the effects of the reference-frame in detail (see for instance, Saraiva et al., 2013) and established that both, reference frame and affective – evaluation are key aspects of interpreting the approach – avoidance behavior in these tasks. In the current study, the participants were asked to adopt a self-referent frame of actions, wherein they were asked to move the manikin toward and away from the target stimuli as if they were themselves moving along the manikin. In this regard as well, our results are in line with previous studies such as Saraiva et al. (2013) where participants are faster to approach than avoid positively evaluated pictures while there wasn’t a significant difference between approaching and avoiding negatively evaluated pictures, the latter in our case was found to be significant (*p* = 0.032). An important point that needs to be highlighted here is that Saraiva et al. (2013) utilized arm flexion and contraction using joysticks as movement indices whereas the task used in the current study use only button presses as in De Houwer et al. (2001) and Krieglmeyer et al. (2010).

Another important caveat for the current study is the fact that our participants are not responding to single specific exemplars that they have formed the “self”- or “stranger” – associations with, as in Sui et al. (2012); rather they have formed the associations with three exemplars that form a cohort based on similarity of features (same colour and shape), and in the approach – avoidance phase of the experiment they are responding to a new cohort of ten colour-shape combinations that either resemble the cohort that was previously associated with the “self” or the cohort that was associated with the “stranger.” It is evident from the participant’s responses across the two categories of stimuli that they were able to generalize the associated response tendencies from a small set of 3 unique exemplars to a larger set of 10 exemplars that were used in the main approach-avoidance task. This is in line with previous proposals, such as made by Sui and Humphreys (2015) where they propose the concept of “self – expansion” implying that it is possible for participants to extend the benefits of “self-association” to other members of the category formed by members that share features. For instance, from a single cyan triangle to all cyan triangles. As mentioned earlier, previous work already demonstrate that participants can generalize response tendencies associated with single exemplars to a cohort of exemplars that has common features with the initially associated shapes. Corollaries of the same have also been illustrated in studies that present the group-prioritization effect as an extrapolation of the self-prioritization effect reported in several studies (Sui et al., 2012; for a detailed review see Sui and Humphreys, 2017). Previous studies cited earlier (Moradi et al., 2015, Enock et al., 2017) have together demonstrated that it is possible for participants to treat cohorts of stimuli as an in-group as opposed to an out-group and accord differential response tendencies to the same on these grounds.

The approach – avoidance task (AAT) itself has also been utilized to study in-group vs. out-group behaviors. For instance, Degner et al. (2016) utilized an automatic approach avoidance task, wherein they were able to demonstrate a significant in-group bias amongst the minority community members, albeit when they were tested in a segregated setup. Similarly, Rougier et al. (2020) demonstrated in-group biases using the Visual Approach/Avoidance Task (VAAST) across both a dominant and non-dominant group. The authors concluded that the approach – avoidance task can be suitable for assessing intergroup attitudes.

The current study extends the findings of both self-expansion and consequent generalization of response tendencies as reported in previous studies (Moradi et al., 2015; Enock et al., 2017) and also the utility of the AAT task for assessment of inter-group attitudes as in Degner et al. (2016) and Rougier et al. (2020). In the current study, the authors were able to find a significantly positive AAT score for the self-related stimuli, while at the same time a negative AAT score for the “stranger – related stimuli.” Given that these findings were true for an entire cohort of “self-related” and “stranger – related” stimuli, it can be argued that the current results also present evidence for a significant in-group bias based on similarity of features between group members.

These findings may have implications in understanding the formation of group – level attitudes for entire groups, based on commonalities and contrasts between group members. Indeed, several studies, point out toward the fact that group identities and consequent attitudes may be formed along the lines of shared characteristics and differences. For instance, Turner et al. (1979) demonstrate that individuals do identify with members of an in-group based on common characteristics and demarcate the members of an out-group based on differences. Similarly, Brewer (1999) also points out that individuals’ behavior toward an in-group may include reservation of positive emotions such as trust or sympathy whereas the lack of the same may manifest in their behaviors toward a perceived out-group.

## Limitations

While the current study demonstrates a clear approach-bias toward a cohort of self – related stimuli, the avoidance bias toward the “stranger – related” cohort may be interpreted with caution. There are several possible reasons for the same, firstly, while there is a bunch of evidence ascribing positive evaluation to self-related stimuli, but the authors did not find a very strong evidence designating negative evaluation to “strangers.” For instance, while Sachschal et al. (2019) provide evidence that patients of posttraumatic stress disorder (PTSD) may be inclined to ascribe negative appraisals to strangers; in another study Vicovaro et al. (2022) demonstrate that the self-prioritization effect can get severely attenuated if the implicit valence associated with the “self” and the “stranger” categories gets reversed by associating them with unpleasant (asymmetrical) vs. pleasant (symmetrical) stimuli, respectively. Hence, the negative attitudes (as indexed by faster avoidance and slower approach) toward the “stranger” label, should be interpreted with a pinch of salt. Finally, it has been pointed out that the participants may have been making a “response toward themselves” by pressing B and “away from themselves” by pressing the Y keys, respectively. Such a pattern may resemble previous studies that involved participants arm movements toward or away from their body; however, we feel that given our participants always had to start from the H key, there is very little probability of the keypresses (B and Y) influencing our results.

## Author’s note

The current study was not supported by any funding agency. The submitted article has not been published previously and is not in consideration for publication elsewhere and all the authors approve its publication in its current form.

## Data availability statement

The original contributions presented in this study are included in the article/supplementary material, further inquiries can be directed to the corresponding author.

## Ethics statement

The studies involving human participants were reviewed and approved by the Institute Human Ethics Committee, Indian Institute of Technology, Kanpur. The patients/participants provided their written informed consent to participate in this study.

## Author contributions

All authors listed have made a substantial, direct, and intellectual contribution to the work, and approved it for publication.
